# Examining the implementation of a community paediatric clinic in a socially disadvantaged Irish community: A retrospective process evaluation

**DOI:** 10.1371/journal.pone.0295521

**Published:** 2024-02-01

**Authors:** Lynn Buckley, Louise Gibson, Katherine Harford, Nicola Cornally, Margaret Curtin

**Affiliations:** 1 School of Public Health, University College Cork, Cork, Ireland; 2 Let’s Grow Together! Infant & Childhood Partnerships CLG, Cork, Ireland; 3 Department of Paediatrics and Child Health, University College Cork, Cork, Ireland; 4 School of Nursing and Midwifery, University College Cork, Cork, Ireland; Marquette University - College of Nursing, UNITED STATES

## Abstract

**Background:**

Understanding interventions and their implementation is essential for improving community initiatives. Kidscope is a community paediatric development clinic providing free health and developmental assessment and onward referral for children aged zero to six years in an urban area of southern Ireland where many children experience complex needs. Established in 2010, Kidscope developed an inter-disciplinary, multi-agency community team by drawing on the strengths of local services and practitioners to deliver holistic approaches to child health and development. Recent studies examining stakeholder engagement and Kidscope outcomes highlighted the need to examine implementation to better understand the processes and mechanisms of the clinic and how events have affected outcomes.

**Methods:**

Guided by the UK Medical Research Council Framework for Developing and Evaluating Complex Interventions, this study used a post-hoc qualitative process evaluation study design with multiple data sources; stakeholder perspectives (interviews, focus group, questionnaires) and document analysis (annual reports, meeting minutes, work plans). A diverse set of research questions were developed in conjunction with a Patient and Public Involvement Group. Guiding frameworks supported thematic analysis of primary data, document analysis of secondary data, and triangulation of findings across datasets.

**Results:**

Data analysis yielded 17 themes and 18 sub-themes. Successful implementation hinged on developing a coalition of linked practitioners and services whose skills were utilised and enhanced within Kidscope to deliver a high-quality healthcare model to vulnerable children and families. Relational and multi-disciplinary working, innovative approaches to implementation and sustainability, training and education provision, and the accessible community location were among the mechanisms of change resulting in improved child, family, practitioner, and system-level outcomes. External factors such as COVID-19 and deficits in Ireland’s disability services posed significant barriers to fidelity.

**Conclusion:**

This study provides evidence of the processes, mechanisms, and model of care employed by a community-based paediatric clinic to successfully engage society’s most vulnerable families and promote health equity. This study makes an important contribution to the field of implementation research by offering an example of a robust approach to conceptualising and measuring implementation outcomes of community healthcare initiative in a changing, real-world context.

## Introduction

The most critical period of human development is from conception to age six years when structures of brain architecture develop [1] which determine child development, well-being, learning and behaviours [2]. Developmental delay; variations in a child’s achievement of expected milestones; can impact children’s ability to participate in activities of daily living, social relationships, and academic performance [3]. Poverty and social disadvantaged can increase risk of developmental delay and poor child outcomes [4]. A considerable proportion of developmental delay is avoidable however [5], with studies highlighting the importance of prevention and early intervention approaches for improving child, family, and community outcomes [6, 7].

Community paediatric clinics provide care to children and families in their locality [8] by treating unwell children, monitoring health and developmental concerns, delivering health promotion services, counselling and advice, and referring to other health and family support professionals [9]. Delivered by a paediatrician and medical team who work in partnership with local agencies, clinics are often implemented in areas of social disadvantage where individuals are increasingly vulnerable [10].

Established in 2010, Kidscope is a community paediatric clinic offering free health and developmental assessment and onward referral for children aged zero to six years living in an urban area of southern Ireland. Kidscope’s catchment area has a history of significant deprivation with several neighbourhoods classed as ‘extremely disadvantaged’ [11]. Many children in the community experience complex needs, ‘an exceptional level of need requiring access to child disability or specialist teams’ [12]. Kidscope gradually expanded into a multi-agency, inter-disciplinary service through significant input from local child and family support agencies [13]. As a complex intervention with several interacting components [14] Kidscope draws on the strengths of local community services and practitioners to deliver holistic approaches to child health and development, different to that of traditional hospital-based paediatric settings [15]. By providing high-quality and timely health and developmental assessment within a vulnerable community, Kidscope aims to intercept the gap within Ireland’s early intervention system [15].

Complex intervention research should consider what is known already and what further evidence would add most to knowledge [16]. Likewise, Marshall (2004) suggests understanding community interventions and identifying approaches to service implementation is essential for improving local services [17]. Kidscope is an established service and several studies have been conducted outlining the value of the clinic for supporting vulnerable children and families [13, 15, 18–20]. Recent studies examining stakeholder engagement and Kidscope outcomes [13, 21] highlighted the need to examine implementation to better understand the processes and mechanisms of the clinic and how events have affected outcomes.

Process evaluations help to understand why a programme is or is not successful, what happens within programmes, and how events affect outcomes [22]. Importantly, process evaluations provide an opportunity to improve prevention and intervention programmes that benefit communities experiencing oppression and marginalisation [23]. Hunt et al. underscore the necessity for process evaluations to reflect the theoretical model on which the intervention is based, to include multiple data collection methods, and to incorporate perspectives of the various individuals involved [23]. The theoretical model on which Kidscope was based, Bronfenbrenner’s ecological systems theory, was the theoretical basis for evaluating Kidscope [24]. This theory assumes that humans encounter different environments throughout their lifespan that influence development and behaviours by organising developmental contexts into levels of influence: family and community environments, services and relationships between services, and local and national policies [24]. Using a process evaluation design, we used multiple data collection methods; perspectives from stakeholders across different levels of Kidscope (internal and external) and across different systems of the child’s environment, as well as secondary sources from linked child and family support services.

### Aim

To retrospectively examine Kidscope implementation to better understand the processes and mechanisms of the clinic and how events have affected outcomes.

## Methods

### Overview of intervention

Kidscope provides health and developmental care and support for children aged zero to six years and their families in a disadvantaged area of southern Ireland. Situated in a health project at the centre of an urban community, the clinic is delivered one day per week and sees approximately 164 children and their families annually, in line with the academic year (September to May). A Consultant Paediatrician from University College Cork delivers Kidscope with support from medical students completing undergraduate community paediatric placements [25]. Kidscope delivery is supported by practitioners across multiple community-based services.

Community Medical Doctors (CMDs); practitioners who assess child health and development, usually in primary care settings, and refer onwards to paediatricians [25]; provide medical support within Kidscope and collaborate with the paediatrician to expedite assessment and onward referral for vulnerable children.

NICHE community health project provides the location for Kidscope. NICHE focusses on improving health, wellbeing, and quality of life for community members [26]. Community Health Workers (CHWs) in the project; health care providers who live in the community they serve to meet unmet health needs in a culturally appropriate manner and improve access to services [27]; use existing relationships with families to support their attendance at Kidscope.

Local Public Health Nurses (PHNs); registered nurses employed by the national health service of Ireland based in community settings and people’s homes [25]; are the main referring agents into Kidscope. PHNs offer administrative assistance, support for family attendance, and provide local knowledge and health and development expertise to Kidscope.

Let’s Grow Together, an area-based childhood (ABC) programme aiming to measurably improve the lives of children (pre-birth to six years) and their families, provides evidence-based groups and targeted services such as Infant Mental Health (IMH) home visiting to families attending Kidscope [28]. The Let’s Grow Together team includes Infant Parent Support Workers, Speech and Language Therapists (SLTs), and a programme-specific PHN who are qualified IMH practitioners. IMH is a relationship-based, trauma-informed approach to supporting families experiencing adversity by building the strengths and capacities of parents and caregivers to best support their children [1]. In addition to providing administrative support and in-clinic expertise, Let’s Grow Together deliver IMH training to Kidscope linked practitioners to build professional capacities to support vulnerable children and families.

Primary care SLTs; practitioners who assess children’s strengths and needs in communication, eating, drinking, or swallowing [25]; support Kidscope delivery by providing in-clinic speech, language, and communication expertise.

Finally, local family support service, Springboard, supports families of children aged four years and over by providing a structured package of care, intervention, and support for parents through counselling (targeting specific symptoms or situations for the parent or family) and psychotherapy (longer-term treatment to understand ongoing issues for the parent or family) [29].

Kidscope’s inter-disciplinary team of linked practitioners is therefore comprised of the Consultant Paediatrician, medical students, and a range of child and family community specialists. To ensure the delivery of high-quality, consistent, and coordinated health and developmental care by the inter-disciplinary team, linked practitioners receive continuous professional development at Kidscope through weekly knowledge sharing meetings, receipt of specialist child health and development knowledge and guidance, and IMH training and coaching. IMH training and coaching consists of a two-day masterclass and monthly network groups delivered by the Let’s Grow Together team to enhance practitioners’ capacity to engage with vulnerable families, particularly in the areas of early social and emotional development [28]. Since establishment in 2010, 73 practitioners have supported clinic delivery, 2,088 children and their families have received assessment, care, and onward referral, and over 2,000 medical students have trained within the clinic [13].

### Study design

We used a post-hoc qualitative process evaluation design [22] with multiple methods; stakeholder perspectives (interviews, focus group, questionnaires) and document analysis (annual reports, meeting minutes, and work plans).

**Fig 1:**
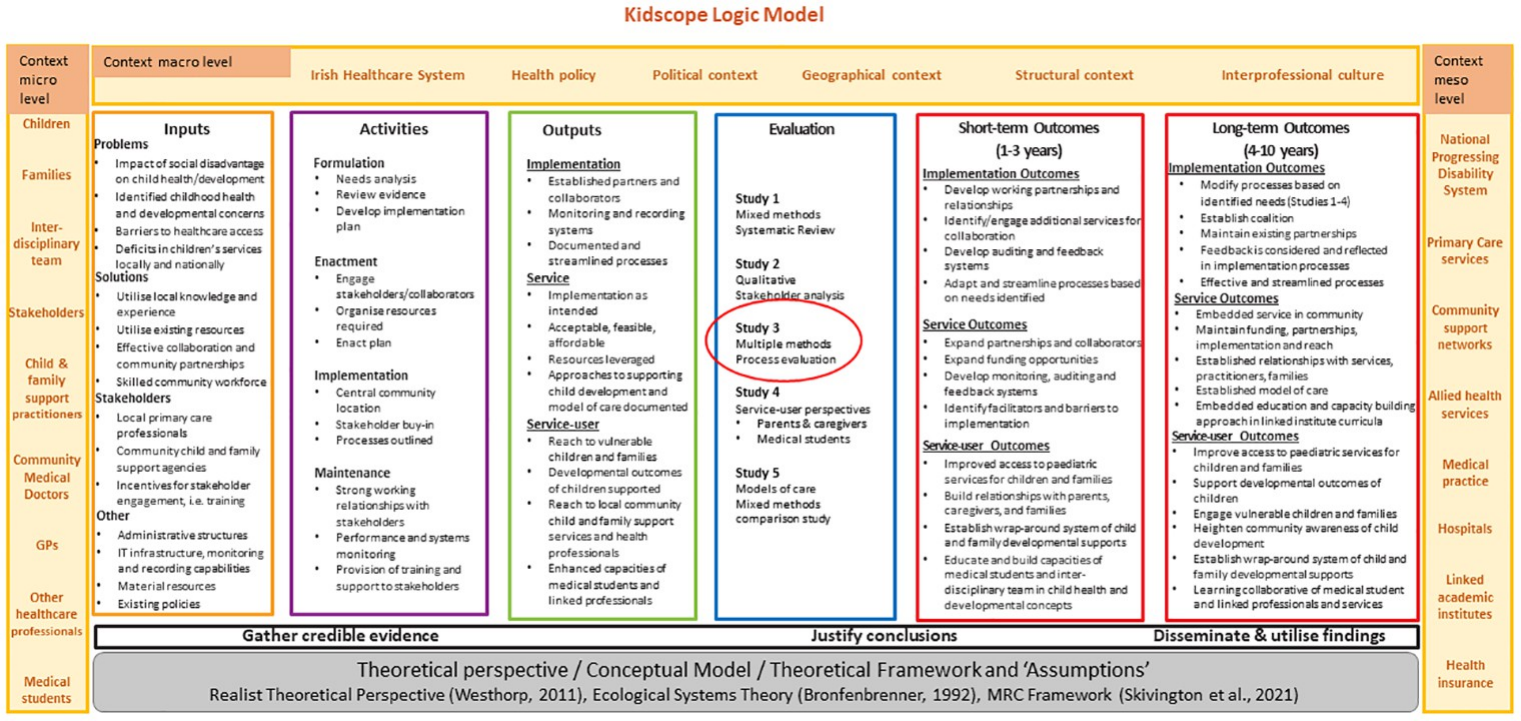
Kidscope logic model.

In order to retrospectively examine implementation processes, the Kidscope Logic Model (Fig 1) informed by Bronfenbrenner’s ecological systems theory [24] provided the theoretical basis for the evaluation and outlined assumptions underpinning the assumed effects of Kidscope (theoretical basis and framework, prevention and early intervention, relational approaches, inter-disciplinary working, child and family-centred care). The Logic Model outlined the inputs, activities, outputs, evaluation approaches, and short and long-term goals, as well as contextual factors influencing implementation.

The UK Medical Research Council Framework (MRC) for Developing and Evaluating Complex Interventions [30, 31] provided the framework through which processes were examined. The MRC facilitated the complex nature of Kidscope and provided a framework of five constructs to examine the intervention (Skivington et al., 2021). Construct 1: *Context*, refers to external factors that influenced the delivery and functioning of Kidscope. Construct 2: *Implementation processes*, refers to the resources and processes through which Kidscope was delivered. Construct 3: *Mechanisms of impact*, refers to how Kidscope activities, and participants’ interactions with them, triggered change. Construct 4: *Fidelity*, refers to the way Kidscope was delivered in both the manner and the spirit in which it was intended. Construct 5: *Outcomes*, refer to the perceived impact of Kidscope and how much of a difference the intervention made [22].

This process evaluation had five main aims:

1. To describe the context in which Kidscope was implemented.
2. To describe the processes involved in Kidscope implementation.
3. To identify the Kidscope activities that triggered change.
4. To examine Kidscope delivery in line with underpinning theoretical model and assumptions.
5. To understand the perceived impact of Kidscope.

### Research questions

Primary and secondary data collection activities (focus group, interviews, questionnaires, and document analysis) aimed to answer a set of questions for each construct examined (Table 1).

1. What external factors influenced Kidscope implementation?
2. What were the processes involved in Kidscope implementation?
3. What were the Kidscope activities that triggered change?
4. Has Kidscope been delivered in line with intended assumptions?
5. What are stakeholders’ perspectives of the impact of Kidscope?

**Table 1:** Process evaluation questions.

Constructs	Elements to be evaluated	Questions
**Context**	Social disadvantage Healthcare policy and systems Healthcare availability Access to healthcare	What factors in the community, social/political context, or other situational issues have potentially affected Kidscope implementation or outcomes?
**Implementation processes**	Engaging core participants Education provision Referrals system Recording, monitoring, and quality improvement Intervention sustainability	What were the procedures for engaging stakeholders? What were the barriers to engagement of individuals, groups, and organisations? To what extent was training provided (consistent with the underpinning theory/assumptions)? How was child and family engagement recorded and monitored? How did continuous improvement occur? How has Kidscope been maintained and sustained?
**Mechanisms of impact**	Collaboration Interpersonal and (inter)professional relationships Setting and structure Approaches to service delivery	How did participants interact with Kidscope? How have stakeholders worked together to implement Kidscope? In what ways has Kidscope engaged vulnerable children and families? What activities/approaches have facilitated Kidscope delivery? What activities/approaches have challenged Kidscope delivery?
**Fidelity**	Kidscope delivery in line with underpinning theoretical model and assumptions Training and education	How has Kidscope effectively engaged and supported vulnerable children and families in their locality? What were the challenges to ensuring fidelity and intended delivery? To what extent was training provided as intended?
**Outcomes**	Systems change Community development Capacity building Workforce education Sustainability	To what extent has Kidscope achieved intended outcomes? How has Kidscope improved the health and developmental outcomes of the community? How has engagement with Kidscope affected the service provision and capacities of linked organisations and individuals? What learnings about Kidscope implementation can inform future engagement and activities?

Adapted from Saunders et al. (2005) [22]

### Sample

To answer the diverse set of research questions, a range of perspectives were required. Twenty-seven stakeholders from medical, academic, primary care, and community and voluntary organisations with previous or current involvement in the implementation of Kidscope participated. In line with recruitment for a stakeholder analysis running concurrent to this study [13], snowball recruitment identified participants. Recruitment began on 1^st^ March 2021. Initial names were retrieved from scoping interviews with five individuals, two involved clinic development and three involved in current implementation. Recruitment continued for 18 months with additional names emerging from the focus group, one-to-one interviews, and questionnaires.

### Data collection

#### Primary data

Primary data addressed all research questions and was obtained through socio-demographic forms completed by participants, scoping interviews (n = 5), a focus group (n = 6), one-to-one interviews (n = 16), and medical student questionnaires (n = 5). The lead researcher (LB) was responsible for conducting the focus group and interviews and collating questionnaire responses. When required, another member (NC) of the working group acted as a non-participant observer. Medical student questionnaires were disseminated via Survey Monkey and a sample were selected at random (MS Excel ‘ = RAND’ function) for inclusion.

#### Secondary data

Participants involved in primary data collection were invited to identify or provide supplementary documentation regarding Kidscope which they felt was pertinent to the research. Secondary data was provided on a voluntarily basis by participants from linked services, services to whom linked practitioners are affiliated, with managerial approval. Most reports were publicly available documents, i.e. annual reports, published on linked service websites. Unpublished materials, namely meeting minutes and workplans, were shared in accordance with GDPR and data sharing best practices, and data which emerged from document analysis were fully anonymised to ensure privacy and confidentiality. Secondary data aimed to address all research questions. Based on the research questions, sources chosen for inclusion were annual meeting minutes (n = 8), annual reports (n = 5), research papers (n = 1), and linked service work plans (n = 4).

### Data analysis

#### Primary data

Audio-recordings and field notes were fully transcribed. Data was collated using NVivo [32]. Thematic analysis involved pooling codes, developing overarching themes and sub-themes, and further review and collapsing of themes [33]. The lead researcher (LB) developed a reflective journal of notes to capture theoretical assumptions prior to interviews and the focus group; thoughts, feelings, and perspectives during and after each interview or focus group; and a reflexive statement considering the researcher’s position within the research and the questions asked. Reflections aided interpretation of the data.

#### Secondary data

Guided by Bowen’s framework, a document analysis of secondary data took place. Documents were analysed iteratively through skimming (superficial examination), reading (thorough examination), and interpretation (thematic analysis) [34]. Analysis of secondary data facilitated the corroboration of findings from primary data analysis and offered additional information not captured via primary data collection.

A second author (MC) conducted a review of codes, themes, and sub-themes developed by the lead researcher. Consensus on meaning was achieved through discussion. Collaboration helped to develop a richer more nuanced reading of the data.

Themes and sub-themes are supported by exemplar quotes denoted by the data collection activity (focus group = FG, interview = I, medical student questionnaire = Q, document analysis = D) and associated participant ID number, i.e. FG-xx, I-xx, Q-xx, D-xx.

### Data triangulation

Guided by Farmer et al.’s triangulation protocol, findings related to the research questions from each data set (primary and secondary) were sorted and collated [35]. Findings were compared with respect to the meaning and interpretation of themes and the number of participants or documents mentioning a theme. Following interpretation of findings across both data sets, similar and unique contributions to the research questions were identified, and a summary of the unified findings for each research question was developed.

### Trustworthiness of data

Trustworthiness of the findings related to credibility, dependability, confirmability, and transferability [36]. The Standards for Reporting Implementation Studies (StaRI) Statement guided the reporting of study findings [37]. Interview and focus group schedules were tested with the research working group (authors) and a Patient and Public Involvement (PPI) Group who piloted schedules through mock interview scenarios. Feedback and suggested edits were incorporated to create final schedules. Concurrent analysis of primary and secondary data facilitated corroboration of findings across data sets, thus reducing the impact of potential biases. To further enhance dependability, two reviewers engaged in transcript review and confirming themes and sub-themes. Themes and sub-theme headings were informed by the Expert Recommendations for Implementing Change (ERIC) to ensure consistent language and descriptions that align with implementation literature for use in isolation or combination with other implementation research [38]. In line with guiding framework [30], a sample of stakeholders (n = 6) reviewed themes and sub-themes during report write-up and provided comments. Feedback ensured credibility and dependability and contributed to study conclusions. To achieve confirmability, excerpts were included to demonstrate how conclusions were drawn. Transferability was addressed by including a detailed description of findings to enable comparisons to be made.

### Ethical considerations

Participants received an information sheet outlining study details. Fully informed written consent was obtained. Ethical approval was received from the Clinical Research Ethics Committee of the Cork Teaching Hospitals, University College Cork, Ireland (reference number: ECM 04/2023 PUB).

## Results

Results are reported in line with the five main research questions. Five codes, 17 themes, and 18 sub-themes emerged from thematically analysing primary and secondary data. Table 2 provides an overview of codes and themes.

**Table 2:** Main codes and themes derived from thematic analysis.

CODE	THEMES	SUB-THEMES
**AIM 1: CONTEXT**	Conceptual and contextual development	History of community
		Developmental delay
		Barriers to healthcare access
		Deficits in children’s services
**AIM 2: IMPLEMENTATION PROCESSES**	Inter-disciplinary team development	Communication and networking
	Involving family members	Empowering parents and caregivers
	Bi-directional referrals process	
	Development of quality monitoring systems	Team meetings
		Auditing and feedback
		Evolving and streamlining
		Safeguarding Kidscope
**AIM: 3 MECHANISMS OF IMPACT**	Multi-agency inter-disciplinary working	Follow-up support and care
	Relational approach	
	Innovative thinking	
	Community setting and infrastructure	
	Openness of parents/caregivers	
	Safeguarding Kidscope	
**AIM 4: FIDELITY**	Model of care	Child and family-centred approach
	Infant Metal Health training	
	Barriers to fidelity	COVID-19
**AIM 5: OUTCOMES**	Child health & development	Holistic child assessment
		Engaging and supporting families
	Systems change	Building a coalition
		Service integration
	Education	Creation of a learning collaborative

### Aim 1: Context

#### Conceptual and contextual development

*History of community*. A large local authority housing development built in an urban community in the 1970s, the area became home to many young families throughout the 1980s and 1990s (D-03, D-12). Poor planning and limited infrastructure were found to be the cause of many social issues in the community,

*“there were no services*. *Houses were poorly built*, *the sewage system was appalling*, *water quality was poor*, *there was no school or shop*. *Young families from all over the city and other areas were thrown together into this situation”*

(FG-04).

The establishment of a family centre in 1980s and a community health project in 1990s aimed to address issues (D-02, D-03). In 2000s, family-based programmes such as antenatal classes, breast-feeding support groups, and parenting programmes were established through local partnerships (D-03). Child health strategies were also introduced,

*“a speech and language capacity building programme educated preschool and school staff on how to recognise speech difficulties and promote speech*, *language*, *and communication”*

(I-09).

These initiatives paved the way for establishing Kidscope (D-01, D-03, FG-04).

*Developmental delay*. Throughout 1990s and 2000s, reports of childhood developmental delay were noted in the area (I-09, FG-04, FG-05). In addition to motor delay and behavioural issues, a significant amount of speech, language, and communication delays were evident,

*“we were continuously hearing of and seeing children with communication needs*. *Speech and language therapy was identified as a significant need in the community”*

(I-09).

Local Early Years practitioners observed deficits in child developmental progress and readiness for Early Years education (I-05). Initiatives to address childhood developmental delay were considered,

*“there were zero supports and services so we looked at how we could change that landscape*. *We chipped away for years*. *Kidscope was a logical next step for children… the gap was there*, *we worked to fill it”*

(FG-04).

*Barriers to healthcare access*. Participants involved in developing Kidscope were cognisant of the barriers families within the community encountered when accessing healthcare,

*“prior to Kidscope*, *families weren’t attending clinical appointments*. *They couldn’t*, *many had no way of getting there*!*”*

(FG-05).

Available healthcare in the community was limited and of lower quality,

*“the health centre in the area was only open certain days for short periods*, *the setting was sub-standard*, *it wasn’t a nice place to go”*

(I-08).

In order to ensure engagement of vulnerable children and families, ease of access was essential,

*“we felt strongly that we needed to base Kidscope locally for people to avail of it”*

(I-09).

*Deficits in children’s services*. Kidscope was delivered in the context of a struggling national disability service providing ad-hoc intervention across Ireland with significant wait times for assessment and intervention (D-02, D-09, D-12), “many children were falling through the cracks” (I-17). Issues remained despite re-structure of the system in 2020,

*“The system is still broken*. *Long wait lists*, *complicated referral forms*, *a new network disability system*, *an old dysfunctional early intervention system…*. *It is difficult for the most able person to navigate so extremely difficult for vulnerable families who are dealing with so much”*

(I-03).

The disjointed system saw children from more affluent areas receive health and developmental services faster through paid private assessment,

*“half of my catchment is in a more privileged area*, *these families are paying for assessments and interventions*, *the divide is very obvious and very sad for the more vulnerable families whose children are left behind by the system”*

(I-12).

Kidscope filled gaps in the national disability service for vulnerable children and families,

*“accessing a Consultant Paediatrician within a matter of weeks… Kidscope is an exceptional system of care for the most vulnerable in our society”*

(I-06).

### Aim 2: Implementation processes

#### Inter-disciplinary team development

The Kidscope team evolved from predominantly medical practitioners to an inter-disciplinary team made up of linked practitioners across medical, primary care, and a range of community services (D-05, D-08, D-13, D-17). In 2010, paediatricians and medical students delivered Kidscope with support from the local PHN department who coordinated clinic appointments and the community health project who appointed one Community Health Worker to assist within the clinic (I-05, I-11, D-05). In 2012, a child and family support service increased its participation through the provision of counselling services for parents and caregivers attending the clinic, and attendance at team meetings (I-11). From 2015, the area-based childhood (ABC) programme provided administration hours and resources to support implementation, and attended team meetings (I-05, D12, D-13, D-14). In 2016, the ABC programme provided additional administration hours, and in 2018 commenced delivery of IMH educational briefings to medical students to enhance their capacity to support vulnerable children and families (I-05, D12, D-13, D-14). From 2018 to 2020, community speech and language therapy provided a dedicated therapist to offer in-clinic speech, language, and communication expertise to families (FG-05). In 2020, the ABC programme’s PHN was appointed to an in-clinic role with a focus on supporting families linked with the ABC programme to attend Kidscope and to register new families (I-05, I-20). From 2021, local CMDs joined Kidscope on a trial basis to provide medical assistance and alleviate the backlog caused by COVID-19. Trial success resulted in the return of CMDs to Kidscope from 2022 (I-21).

*Communication and networking*. Successful implementation was achieved through strong communication and networking by the inter-disciplinary team and linked developmental supports (I-10, I-11, FG-01, FG-02, FG-03, FG-05, D-07, D-08, D-16),

*“these connections sum up why Kidscope has been so successful*. *Everyone in the Kidscope circle is communicating and working together*, *with one goal”*

(I-21).

Effective and consistent communication between the referring PHN, the PHN department administrator, and Kidscope administrator ensured successful coordination of clinics (I-06, I-07, I-18, I-22). Working relationships developed between linked services ensured high-quality follow-up support and care (I-10, I-11, FG-01). Communication between linked practitioners and clinicians facilitated a more thorough understanding of children’s situation and needs,

*“any deficits in the students’ and my history of the family is supported and filled in by the interdisciplinary team”*

(I-22).

#### Involving family members

*Empowering parents and caregivers*. Support, guidance, and advice provided during Kidscope consultations and follow-up care empowered families to become actively involved in children’s health and development (I-06, I-09, I-15, I-19, I-21, I-22),

*“we do our best to provide parents with the knowledge to become active agents in their child’s health and development”*

(I-21).

SLTs *“modelled”* language promoting strategies for families within consultations to ensure successful implementation in the home setting (I-13). Additional time was allocated to discuss children’s needs and care plans with parents and caregivers,

*“giving that extra bit of time in the consultation to really understand the advice and plan is so important*, *it sets them up for more positive results”*

(I-21, I-22).

#### Bi-directional referrals process

PHNs were the main referrers into Kidscope,

*“PHNs are the first point of contact for families in the community and refer any child with a query regarding health or development”*

(I-22).

After clinic consultations, the inter-disciplinary team referred children and families onwards to range of services including primary care, early intervention, and child and family support agencies (I-13, D-1). Referrals from Kidscope were perceived to be of higher significance owing to the inter-disciplinary nature of assessments and accompanying reports,

*“referrals from the clinic are often picked up faster as they receive more detail and appropriate information to satisfy service’s acceptance criteria”*

(I-19).

In a bi-directional approach, services re-referred children and families back to Kidscope when needed,

*“as the child develops*, *something else might crop up for them which requires additional assessment”*

(I-15).

As a direct result of engaging with and supporting Kidscope from 2021, and in a novel approach, CMDs began to refer children from their own community clinics into Kidscope when a more specialised opinion was required,

*“they refer children with an issue that they feel is not resolving and for which specialist care from a paediatrician is needed”*

(I-21).

#### Development of quality monitoring systems

*Team meetings*. Inter-disciplinary team meetings took place with linked practitioners after every Kidscope session to discuss the children and families who attended the clinic and to develop care plans(I-03, I-04, I-8, I-15, I-22, Q-2, Q-3, Q-4, FG-01, FG-02, FG-05). Medical students presented findings and provided summaries to the team,

*“it’s a valuable learning opportunity to share with the wider team their notes*, *birth and social histories*, *health and developmental concerns*, *and next steps for the child and family”*

(I-14).

Paediatricians found inter-disciplinary team meetings to be essential for obtaining additional information from linked practitioners about the families attending Kidscope,

*“as they are the eyes and ears on the ground*, *they are able to fill in the blanks that we may not have been able to during consultations about social*, *family*, *or environments factors which could be influencing children’s’ development”*

(I-22, D-05, D-08).

*Auditing and feedback*. Service quality was reviewed at bi-annual implementation meetings (D-03, D-04, D-05, D-06, D-07). Clinic needs were identified through discussion,

*“we discuss issues related to clinic implementation*, *challenges to engagement*, *and plans for clinic development and sustainability”*

(I-22).

Flexibility and adapting to needs ensured service quality was maintained,

*“since the early stages*, *we made a conscious effort to listen to everyone involved in the clinic*, *and we used feedback to adapt and to ensure the service meets the needs of everyone involved”*

(I-14).

Social issues and community needs were often highlighted by families during consultations (D-04, D-16, D-18). Based on feedback, the inter-disciplinary team adjusted the service to ensure children and their families received appropriate care and support,

*“we began to see more presentations of gross and global developmental delays as many children were developing and growing in temporary accommodation hubs*. *The effects on children’s development were evident*, *we needed to consider this and offer additional layers of support”*

(I-21).

*Evolving and streamlining*. Implementation processes evolved throughout the twelve-year period, “we’re always finding ways to improve as an inter-disciplinary team” (I-21). In 2012, after-clinic team meetings were developed as a direct result of difficulties sharing information with referring PHNs and the inter-disciplinary team,

*“it made sense to use time after clinics to meet as a group*, *share findings*, *and develop child and family care plans”*

(I-20, D-02, D-07).

From 2015, linked services appointed a designated staff member to ensure a consistent link with Kidscope and consistent feedback to their wider team (FG-01, I-06, I-13, D-13, D-17). Administrative processes continuously evolved (I-05, I-13, I-20, I-21, D-12, D-13, D-14). The appointment of a dedicated administrator from 2016 resulted in more effective administrative processes (I-05, 1–15, D-09, D-13). The introduction of a SLT in 2018 and a partnership with CMDs in 2022 highlighted Kidscope’s efforts to streamline community paediatric services in the community,

*“we were doing so much work that was similar*. *By coming together*, *baseline assessments are now completed by multiple disciplines in Kidscope*, *expediting the process for children”*

(I-22).

*Safeguarding Kidscope*. Processes to safeguard the Kidscope service were common practice and involved predominantly local individuals and services,

*“we cannot underestimate the undertaking it is*, *and the resources needed to keep it running*, *it really is the good will of those involved that keeps Kidscope going”*

(I-16).

Lack of funding and support from larger organisations and linked institutions resulted in continuous efforts by locals to ensure continued delivery,

*“the clinic doesn’t receive specific funding apart from small pots of funding from local services for their staff to engage*. *It is a small group effort and good will from a lot of the services that it even still exists”*

(I-08).

Participants voiced the need for guaranteed and dedicatedfunding to ensure clinic sustainability,

*“It is such a special clinic; we need to mind it and sustain it as best as possible for the children and families in this area who need it most”*

(I-20).

### Aim 3: Mechanisms of impact

#### Multi-agency inter-disciplinary working

Kidscope evolved into a multi-agency, inter-disciplinary service with a core goal of supporting the needs of children and families (I-06, I-11, I-20, I-21, D-07, D-09, D-12, D-15, D-17),

*“No one service can do it all*. *When you have multiple services and disciplines linking together all with the same goal*, *it reduces workload and improves health outcomes”*

(FG-01).

The inter-disciplinary team’s collective knowledge of families added to clinicians’ interpretation of children’s needs and developmental contexts,

*“their knowledge of families’ social and environmental situations has become invaluable and has really moulded the Kidscope model”*

(I-22).

This approach was compared to other paediatric healthcare models,

*“you have extra information than you get in a hospital clinic*, *having those links in the community to professionals already working with the family is just so useful”*

(I-16).

#### Follow-up support and care

Kidscope’s interdisciplinary team acted as a network of support for children and families before, during, and between clinic appointments. The wrap-around system of care began when families entered the clinic,

*“community health workers and administrators often help families with literacy issues to complete developmental questionnaire”*

(I-09).

Community health workers assisted families with completion of referral forms,

*“forms are lengthy and complicated*. *If the paediatrician feels they need help*, *they give us the nod and we go through everything with the family”*

(I-02).

Likewise, the in-clinic PHN supported families after consultations with additional queries,

*“I often take families into another room after consultations or ask if they would like a call when they have processed everything”*

(I-21).

To ensure effective implementation of child and family care plans, linked practitioners provided follow-up care and advice between appointments,

*“it’s a huge effort by community practitioners*. *Families are supported as best as possible and return to Kidscope to continue the care they need”*

(I-19).

#### Relational approach

A relational approach to working with children and families was adopted by Kidscope practitioners (FG-05). This encouraged openness and communication with parents and caregivers, thus achieving a better understanding of the child’s health or developmental issue,

*“grounding the family’s experience in supportive relationships helped to gain better insight into what’s really going on for the child”*

(I-08).

Relational working was not confined to in-clinic interactions, administrators also discussed using this approach with families,

*“they often describe their struggles when I call to organise the appointment*. *It can be very intense*. *I listen and reassure them that attending Kidscope will be beneficial”*

(I-07).

#### Innovative thinking

Implementing a novel paediatric clinic in the community hinged on innovative and joined-up thinking,

*“without the passion*, *knowledge*, *and innovation of these individuals*, *Kidscope would not have come about”*

(I-11).

Securing buy-in from a mix of local initiatives such as the community health project and PHNdepartment was described as a *“pioneering”* (FG-04). Input from the local child and family support service and ABC programme was driven by,

*“a passion for improving child and family outcomes in the area and thinking outside of the box on how best to achieve this”*

(I-18).

Partnerships with community speech and language therapy and CMDs marked a novel and innovative approach to improving the Kidscope service and streamlining healthcare approaches in the community,

*“we are already seeing more joined up thinking because of the Community Medical Doctors and Speech & Language Therapists coming into the Kidscope model and seeing how services can work together to be of most benefit for the community”*

(I-21).

To ameliorate against challenges posed by limited funding streams, resourcing constraints, COVID-19, and clinic waitlists, new ways of working ensured clinic longevity,

*“combined efforts and thinking outside of the box has been a common feature of Kidscope’s success”*

(I-08).

#### Community setting and infrastructure

The Kidscope setting was found to be a driver for change due to its important role in engaging families,

*“families felt comforted and supported–this was pivotal to gain their trust and to encourage continued attendance”*

*(I-08)*.

The clinic’s *“relaxed”* and *“homey”* nature was credited for facilitating increased communication with parents and caregivers, helping to elicit thorough family histories and capture greater insight into the context of the child’s development (I-13, I-21, Q-02). Furthermore, paediatricians noted the value of the setting for conducting child assessments,

*“the corner stone to developmental assessment is observation*. *Children can walk*, *run*, *play*, *stack blocks*, *and talk and draw here at Kidscope*, *they do that because they are relaxed”*

(I-22).

Kidscope location in the centre of the community ensured families were able to access additional supports from the health project between appointments (I-16, D-03, D-12, D-13).

*Openness of families*. The openness of families was a significant driver for change and contributed to the success of Kidscope,

*“without families’ trust in the service and being so open and sharing their stories*, *the service would not be as effective”*

(I-02).

Parent and caregiver commitment to children’s health aided the history-taking process and helped develop trusting relationships with clinicians,

*“In the consultations there is real and powerful story telling*. *This can be very hard for families*. *I find this is the place where relationships really develop between the family and Kidscope team”*

(I-21).

Equally, families’ willingness to learn strategies to improve child health and development and to advocate for children’s needs was a significantly aided the effectiveness of Kidscope,

*“when you have parents come on board in the way families at Kidscope do*, *it is such a great help for us as practitioners”*

(I-20).

### Aim 4: Fidelity

#### Model of care

*Child and family-centred approach*. Kidscope was implemented with fidelity to the underpinning framework, assumptions, and intended goals through adopting a child and family-centred model of care (I-07, I-21, I-22, D-05, D-06, D-07). This model allowed the inter-disciplinary team to examine the child’s engagement and interactions with the range of systems influencing their health, development, and well-being (I-15, I-19, I-21, I-22),

*“Keeping the child at the centre of our work*, *we looked at ways to support and influence the systems around the child”*

(I-21).

Successful engagement and collaboration with parents and caregivers facilitated a better understanding of the child’s social, family, and medical histories resulting in the provision of more appropriate and effective care (FG-01, FG-02, FG-05, I-15, I-19, I-21, I-22).

*Infant mental health training*. Implementing an IMH training programme within Kidscope ensured linked practitioners were educated on the underlying assumptions of Kidscope, trauma-informed practices and evidence-based approaches to best support vulnerable children and families (Q-1, Q-2, Q-3, Q-4, Q-5, D-11, D-12, D-13). In addition to knowledge uptake on child health and developmental concepts, the training also provided inter-disciplinary team members with a common language through which they could discuss children’s social and emotional needs and the parent-child relationship, ensuring consistency across the team and fidelity to the service model,

*“we all came from different disciplines and backgrounds*, *some medical and others community*, *so having that language gave us a way to communicate and ensured we were on the same page”*

(I-13).

#### Barriers to fidelity

*COVID-19*. COVID-19 posed the most significant barrier to sustainability and fidelity to service delivery. From 2020 to 2022, nationwide lockdowns and the closure of healthcare services impacted service continuation (I-15, D-15, D-16),

*“We tried to adapt and offer the service as best we could*. *We were so aware of the needs of families and how disruption to healthcare access and social and developmental supports would have significant consequences for children*, *it was a scary time”*

(I-15).

Health and safety measures including face masks, two-meter distancing, and restrictions on numbers within the clinic directly impacted Kidscope’s model of care,

*“the relational approach we spent so long developing had to take a back seat*, *it was a challenge for us all but mainly the children and families”*

(I-07).

Telehealth calls were introduced as a “*means to an end”* (I-21), and were accompanied by challenges,

*“we tried telehealth calls and phone reviews*, *these went well but it wasn’t the same as being face-to-face and being able to have those chats in person and to really observe the child”*

(I-13).

### Aim 5: Outcomes

#### Child health & development

*Holistic child assessment*. Child health and developmental assessments were medical student-led, supervised and supported by a Consultant Paediatrician, and often involved the in-clinic PHN and SLT (I-13, I-16, I-21, I-22, Q-2, Q-3). This approach facilitated high-quality and holistic assessment of the child,

*“the work isn’t each discipline in isolation*. *Disciplines work together to assess and implement a child and family care plan*. *We are a group of different professionals working together for one goal*, *the benefit of the child”*

(I-16).

The in-clinic PHN supported health and developmental observations,

*“I support the medical students in this process*, *initiate conversations with the caregivers if required*, *play with the child and model to students how best to approach observations”*

(I-21).

Paediatricians explored the detail obtained by medical students, observed the child’s interactions, and engaged one-to-one also, *“in this way I model to the students how best to conduct assessments”* (I-22). The SLT completed baseline profiles and provided specialist feedback to parents and caregivers regarding children’s speech, language, and communication needs,

*“I look at social interaction skills*, *play skills*, *understanding of language*, *use of language*, *and speech”*

(I-13).

*Engaging and supporting families*. Effective support of children was achieved through successful engagement and support of families, *“Kidscope supports and addresses the needs of children by also supporting the needs of families”* (I-01). Relationships were developed with families to promote engagement and encourage re-attendance,

*“Prior to Kidscope*, *vulnerable families felt discouraged from attending clinical appointments*. *At Kidscope it was very important to put them at ease*, *make them comfortable*, *and build trust”*

(I-17).

Through Kidscope, parents and caregivers received counselling, parenting support, and capacity building programmes delivered by linked services, (I-01, FG-4, Q-5, D-08). Families were supported to attend appointments,

*“we are involved in reminding families of the date and time*, *accompanying them to the clinic if needed*, *and helping to enact the care plan”*

(FG-02).

Community health workers assisted with completion of developmental questionnaires and provided post-consultation support (I-07, D-05, D-06, D-07). PHNs ensured children and families were supported in-between appointments,

*“It’s such great piece of mind that a community health professional can pop out to visit them sooner than we can see them again in clinic”*

(I-11).

#### Systems change

*Building a coalition*. A coalition of community-based practitioners developed through Kidscope (I-05, I-11, I-15, I-19, I22, FG-01, FG-05, D-08, D-12, D-15). Working relationships cultivated in Kidscope between services and practitioners were nurtured through collaborative provision of follow-up care and support to children and families across the community (I-05, I-13, I-19),

*“we were all working in our own silos before*. *Now we work together to deliver a diverse package of support*, *not just to families engaged with Kidscope*, *we discuss and share knowledge about how best to support children and families across the community”*

(FG-01).

The coalition also consisted of hospital-based paediatricians who provided support and guidance to linked practitioners delivering follow-up support and care between Kidscope appointments (I-15, I-22). Regular recruitment onto the inter-disciplinary team ensured expansion and inclusion of new disciplines,

*“at review meetings we explored how best to recruit new members*. *An example of this is the introduction of Speech and Language Therapists and Community Medical Doctors*. *It didn’t happen instantly*, *but we worked hard to try to develop the expertise on the team”*

(I-22).

*Service integration*. Collaboration with Kidscope aided the integration of similar ways of working and approaches to supporting children and families across community services,

*“the clinic was a real driver in bringing us together and aligning our ways of working to support children and families”*

(I-06).

Kidscope contributed to the implementation of new initiatives offered by linked services (D-03, D-06, D-11, D-12, D-18),

*“because of the increasing number of new families coming into the centre through Kidscope*, *we were able to apply for funding to run new groups such as parent support groups and postnatal depression services”*

(I-17).

Kidscope also directly contributed to the development of a new child and family support service,

*“our programme grew out of Kidscope and became a service that could provide the supports families needed which were identified through Kidscope”*

(I-15).

#### Education

*Creation of a learning collaborative*. Educational offerings and IMH training resulted in the development of a learning collaborative among linked practitioners (FG-01, FG-02, FG-05, I-06, I-08, I-11, I-20, Q-01, Q-02, Q-03, Q-04, Q-05),

*“we learn so much from the paediatrician*, *this impacts our whole way of working with other families in the community too”*

(FG-01).

A system of sharing specialist knowledge and expertise at after clinic meetings was found to aid and inform practitioner service provision to the benefit of families attending Kidscope and those across the community (FG-01, FG-02, FG-05, I-06, I-08, I-11). Training within Kidscope shaped medical students’ learning and perspectives of paediatric healthcare models, particularly those best suited to supporting vulnerable communities,

*“through working with other disciplines in the community*, *we learned it was possible to holistically address developmental concerns”*

(Q-03).

## Discussion

### What external factors influenced Kidscope implementation?

Findings show Kidscope was implemented in the context of a disjointed early intervention system throughout the 2010s, replaced by a struggling network disability system from 2019. Although healthcare policies at the time referenced “equitable access” and “integrated healthcare within communities” [39, 40], many of these promises were not realised on the ground. The *Better Outcomes Brighter Futures National Policy Framework for Children and Young People* promised investment in the early years, and in prevention and early intervention for vulnerable groups [39]. However, in 2016 the HSE reported “wide variation in services resulted in some children and families falling through the cracks or having little or no access to services” [41]. The *National Policy on Access to Services for Children & Young People with Disability & Developmental Delay* stated, “no child will be left without timely access to an appropriate service to meet their needs” [42]). By year end 2022, 18,000 children with disabilities awaited initial contact by the Network Disability Team [43] and 110,000 children were waitlisted for primary care services [44].

The gap in the system for disadvantaged families without the means to access services widened [15]. Significant wait-times and difficulties accessing mainstream services placed pressure on Kidscope, posed risk to service delivery and its model of care, and threatened the provision of health and developmental care for society’s most vulnerable children and families. Adaptability, flexibility, and innovative thinking facilitated Kidscope’s interaction with this changing and pressurised context.

### What were the processes and mechanisms of change involved in Kidscope implementation?

Successful implementation of Kidscope was found to hinge on collaborative processes and strong working relationships. Development of a multi-agency, inter-disciplinary team facilitated a wrap-around system of care for vulnerable children and families. Effective communication and networking resulted in the development of comprehensive child and family care plans and follow-up support for vulnerable children and families in their locality. Findings align with previous research on the importance of collaborative inter-professional teams for making complex and important healthcare decisions, particularly for vulnerable populations [45].

Relational working facilitated the engagement of vulnerable families. Lewing et al. highlight the importance of supportive and trusted practitioner-child relationships as a component of family-centred interventions designed to support vulnerable children [46]. Likewise, Boshoff et al. show trusting and constructive relationships between health practitioners and parents of vulnerable children can have a lasting influence on future relationships with support services [47]. Adding to the evidence-base, our findings highlight the additional value of relational working for empowering parents and caregivers to become active agents in their child’s health and advocates for their developmental needs.

Engaging in quality assurance processes such as team implementation and bi-annual review meetings were found to capture stakeholder voices and identify service needs. Adding to the evidence offered in previous evaluations of similar clinics [3, 48, 49], our findings underscore the importance of flexibility, adaptability, and innovation in ameliorating against challenges such as limited funding streams, resourcing constraints, COVID-19, and clinic waitlists.

### Was Kidscope delivered in line with intended assumptions?

Kidscope’s service model evolved concurrent to the development of the multi-agency, inter-disciplinary team. The development of a child and family-centred model of care ensured fidelity to the intervention’s underpinning framework and assumptions by providing high-quality and effective support for vulnerable children. Maile et al. highlight the need for social paediatrics to adopt an approach where the child is viewed within their social context and care is therefore facilitated through collaboration among these contexts [50]. Argal et al. argue family-centred models of care are mostly broad and conceptual, with less clarity on how to translate theory into practice [51]. Our study offers insights into the processes that have successfully accompanied and aided implementation of a child and family-centred model of care in a disadvantaged community, namely the importance of engagement and empowerment of parents and caregivers.

### What are stakeholders’ perspectives of the impact of Kidscope?

Kidscope was found to contribute to systems change at child, family, community, and academic levels. Child health and developmental assessments were conducted in a holistic approach by the multi-agency, inter-disciplinary team which heightened the quality of observations, improved effectiveness of child and family care plans, and increased appropriateness of onward referrals. Shipley et al. confirm the value of inter-disciplinary teams who understand child health in the context of their community and have the leadership and collaborative skills to improve the health of children in their communities [52].

Development of a coalition of linked practitioners and implementing activities to promote growth of the coalition facilitated a system of community support from a diverse set of expertise working in unison to support vulnerable children and families. Partnering with primary care services marked an innovative way of working and streamlining services to expedite pathways to assessment and intervention. Findings align with previous research which highlighted the value of improving quality of care and promoting healthcare systems change through community-based partnerships [53, 54].

Educational offerings within Kidscope enhanced linked practitioners’ knowledge of specialist early years health and developmental concepts, improving professional practice in the Kidscope catchment area and wider communities. IMH training provided inter-disciplinary team members from various backgrounds and disciplines with a common language to discuss and understand the parent-child relationship and children’s social and emotional needs. Martin et al. highlight the value of IMH training for enhancing practitioner knowledge of developmental stages and increasing their confidence and competence in supporting vulnerable parents [55]. Finally, exposure to a community paediatric clinic influenced undergraduate medical student training through experiential and transformative learning. Findings support the replication of this training model across medical fields to the benefit of the wider community [15]. Given the significant contribution of PHNs to Kidscope implementation and the value of Kidscope for their learning and professional development [12], consideration should be given to extending training opportunities to undergraduate nursing students to gain exposure of such an effective community-based model of paediatric healthcare.

Paediatricians’ work within Kidscope was enhanced by collaborative partnerships. Community-based linked practitioners’ knowledge of families’ social histories provided paediatricians with a more nuanced understanding of the child’s developmental context, resulting in more appropriate and effective child and family care plans. Summarising the value of such a whole community approach, Ukpeh concludes, “the social determinants of health go beyond medical care; partnerships with other professions serving children and youth, cultural sensitivity, and advocacy are ingredients for optimum health” [[8, p.301].

Finally, engagement with Kidscope facilitated the integration, expansion, and development of other community services. Relational working approaches established in Kidscope infiltrated wider community-based settings and systems, influencing approaches to working with vulnerable children and families across the community.

## Implications

This study adds to the literature on community child health clinics by offering insights into the processes and strategies required for successful implementation in a changing, real-world context. This study also offers an example of a successful model of care for engaging vulnerable families while also ensuring fidelity, success, and longevity. Equally, findings can make an important contribution to the field of implementation and approaches to enhance efficacy in implementation research. Proctor et al. highlight the concept of “implementation outcomes” and the importance of research focussed on conceptualising and measuring implementation to pave the way for studies focussed on the comparative effectiveness of implementation strategies [56].

Findings can be used by policymakers to sufficiently fund and sustain Kidscope. McCosker and Matan report detrimental impacts to the implementation of community-based initiatives as a result of limited funding [57]. In line with our findings, the authors also highlight the reliance placed on individual members or organisations because of limited external funding [57]. Good will does not sustain services indefinitely. Linked academic institutes and hospitals must recognise the role Kidscope plays in educating future physicians and paediatricians, its value to local practitioners and services, and its contribution to child, family, and community outcomes. Such a valued and effective intervention must be cared for and supported appropriately.

More broadly, findings promote use of a child and family-centred model of paediatric healthcare across disadvantaged communities in Ireland. To see true alignment with national policies, effective and integrated healthcare can be achieved through implementation of community paediatric clinics in communities where existing local knowledge and expertise can be utilised. Findings from this study, together with additional research on Kidscope [12, 15, 19], provide a template for implementation of a community paediatric intervention which can significantly benefit the most vulnerable in society.

## Conclusion

This study evaluated the processes involved in implementing a community paediatric clinic in a disadvantaged Irish community. Findings provide useful insight into the processes and mechanisms which have aided or posed threat to implementation. Building and sustaining a community coalition of child and family practitioners, and an effective model of care to support vulnerable children and families in their locality were among the main contributors to clinic success. Relational working engaged harder to reach families and provision of a training and education strategy enhanced the capacities of linked practitioners and their affiliated service to the benefit of children and families, therefore ensuring service fidelity. Significant gaps in Ireland’s disability services and early intervention system underscore the real and current need to reform paediatric healthcare policy in Ireland. Findings provide a comprehensive overview of the implementation of an alternative approach to paediatric healthcare. By utilising existing community supports and expertise, high-quality paediatric healthcare can be made available to society’s most vulnerable children and families in their locality. Findings can inform future health policy decisions regarding the implementation of community paediatric clinics in Ireland and elsewhere.
